# Biofilm Formation and the Pore Proteins *ompF* and *ompC* Lead to Heteroresistance in *Escherichia coli*

**DOI:** 10.3390/antibiotics15070705

**Published:** 2026-07-20

**Authors:** Jiaojiao Gao, Yating Wu, Xianlan Ma, Xiaowei Xu, Zhijie Zhang, Wei Shao, Nan Zheng, Tongjun Guo, Yankun Zhao

**Affiliations:** 1 Ministry of Agriculture and Rural Affairs-Laboratory of Quality and Safety Risk Assessment for Agro-Products, Key Laboratory of Agro-Products Quality and Safety of Xinjiang, Institute of Quality Standards & Testing Technology for Agro-Products, Xinjiang Academy of Agricultural Sciences, Urumqi 830091, China; 320222682@stu.xjau.edu.cn (J.G.); yatingwu@xaas.ac.cn (Y.W.); 320242735@stu.xjau.edu.cn (X.M.); 320232680@stu.xjau.edu.cn (X.X.); 320222697@stu.xjau.edu.cn (Z.Z.); 2 Xinjiang Meat and Milk Herbivore Nutrition Laboratory, College of Animal Science Xinjiang Agriculture University, Urumqi 830052, China; dksw@xjau.edu.cn; 3 Key Laboratory for Quality and Safety Control for Milk and Dairy Products of Ministry of Agriculture and Rural Affairs, Institute of Animal Science, Chinese Academy of Agricultural Sciences, Beijing 100193, China; zhengnan_1980@126.com; 4 Feed Research Institute, Xinjiang Uygur Autonomous Region Academy of Animal Sciences, Urumq 830091, China; guotuoxj@sina.com

**Keywords:** *Escherichia coli*, heteroresistance, pore proteins *ompF* and *ompC*, biofilm formation

## Abstract

*Escherichia coli* (*E. coli*) is a major pathogen responsible for mastitis and calf diarrhea in dairy cows and has developed heteroresistance (HR) to a wide range of antibiotics. An increasing number of animal studies have shown that the presence of heteroresistance leads to failure of antibiotic therapy. The aims of this study were to investigate whether heteroresistance exists in *E. coli* and to investigate the biological characteristics of heteroresistant *E. coli* and its resistant subpopulations in order to investigate possible heteroresistance mechanisms. In this study, we screened heteroresistant *E. coli* by the Minimum Inhibitory Concentration (MIC) test, Kirby–Bauer (K-B) test, and population analysis profile (PAP), and analyzed the heteroresistance bacteria by a combination of resistance stability test, growth curve test, biofilm formation ability test, transcriptomics and qRT-PCR characterization and mechanism. The results of the K-B test, MIC test and PAP experiments showed that the strains D2, D8, D14, D15, and D19 were heteroresistant to amoxicillin/clavulanic acid (AMC), and the frequency of heteroresistant subpopulations ranged from 3.95 × 10^−6^ to 8.11 × 10^−5^. The resistant subpopulation resistance of strain D2 was stable and there was no growth lag. The results of transcriptomics testing confirmed that the heteroresistance mechanism involves *ompF*, reduced or absent *ompC* pore protein expression, and increased biofilm formation. This finding provides new insights into the molecular regulatory pathways involved in the development of heteroresistant strains of *E. coli*.

## 1. Introduction

*E. coli* is a Gram-negative conditionally pathogenic bacterium that is ubiquitous in nature [1]; it is one of the main causative agents of persistent and recurrent mastitis in dairy cows [2,3]. Mastitis is a common disease in dairy cows that, if not prevented and treated in a timely and effective manner, will lead to a decrease in milk yield and quality, which in turn will trigger significant economic losses [4,5]. Currently, the prevention and control strategies of *E. coli* infection in animals have long relied on antibiotic treatment. As a zoonotic pathogen, *E. coli* is highly transmissible and has a rapid development of drug resistance, capable of causing clinical diseases that affect humans and animals of different ages [6]. In human medicine and animal husbandry, resistance of *E. coli* to a wide range of antibiotics is rapidly increasing and this problem has been widely documented globally [7]. The irrational use of antibiotic drugs in veterinary medicine has led to increasing resistance in *E. coli* [8]. At the same time, under the pressure of sustained action of different types of antimicrobial drugs, binding and transmission of drug-resistant plasmids occur between strains, leading to multiple drug resistance (MDR) [9,10,11,12]. The increase in drug resistance and the emergence of MDR strains pose a major threat to the control of *E. coli* disease and public health safety. It has been found that *E. coli* also has another type of resistance, namely bacterial heteroresistance (HR).

Heteroresistance refers to the presence of subpopulations of a single isolate that differ in their susceptibility to a particular drug in their cultured populations, i.e., some subpopulations are susceptible to the drug while other subpopulations exist that are resistant to the drug [13,14,15]. The selection of ‘sensitive’ drugs that are screened according to the broth dilution method recommended by the Clinical and Laboratory Standards Institute (CLSI) in the United States results in the inability to completely eliminate some subpopulations of heterogeneously resistant bacteria, which can then develop into subpopulations of resistant bacteria [16]. The result is that some resistant subpopulations of heterogeneously resistant bacteria cannot be completely eliminated and develop into fully resistant strains. Heteroresistance is an intermediate stage from drug resistance to clinical resistance, and it is also one of the main reasons for the failure of clinical antimicrobial therapy. In recent years, more and more animal experiments have demonstrated that the presence of a small number of resistant subpopulations in a bacterial population can lead to antibiotic treatment failure and may be associated with disease treatment failure, which in turn poses a great potential threat to human health [17,18]. Stable heteroresistance results from stable genetic mutations in resistant subpopulations [19], altered expression of nonspecific resistance genes [20], and growth-adaptive costs of resistant subpopulations [21] that do not differ significantly from those of the dominant subpopulation; unstable heteroresistance results from mutations in resistance genes that induce other compensatory mutations [22] and from non-stable resistance mutations in the bacteria themselves [23]. Recently, it has been shown that heteroresistance is also associated with biofilm formation. Highly complex interactions within the biofilm, such as spontaneous mutations, external stimuli (e.g., host immune system, antimicrobial therapy), internal microenvironment (oxygen, nutrients, and pH gradients), and interspecies interactions, can lead to the formation of broadly different subpopulations within the biofilm layer, and the higher the biofilm biomass the more pronounced the interactions are, and the greater the number of emerging subpopulations will be [24,25]. In *E. coli* colonies, active growth cells are located in the lower layer, whereas non-growth starved bacteria inhabit the upper layer in response to diffusion-based nutrient supply, and it is inferred that biofilm formation may also be related to heterogeneous drug resistance in *E. coli* [26].

β-lactam antibiotics, due to their widespread use in clinical medicine and animal husbandry, have become the mainstay of treatment for diseases caused by *E. coli* [27]. Heteroresistance studies against *E. coli* have included a variety of β-lactam antibiotics such as benzoxacillin, cefotifen, ceftazidime, ceflorin, cefepime, ampicillin/sulbactam, piperacillin/tazobactam, as well as carbapenem antibiotics. Amoxicillin/clavulanic acid (AMC) is one of the most commonly used antimicrobials in many countries [28,29], and its mechanism of action and resistance studies are important for understanding the resistance mechanisms of *E. coli*. AMC is a combination antibiotic consisting of amoxicillin and clavulanic acid, where amoxicillin is a broad-spectrum β-lactam antibiotic, whereas clavulanic acid is a β-lactamase inhibitor that protects amoxicillin from degradation by β-lactamases produced by bacteria [30].

Heteroresistance of *E. coli* to AMC has not been well characterized despite several studies on the mechanism of bacterial resistance to β-lactams. The aim of this study was to investigate the characterization and mechanism of heteroresistance of *E.coil* isolated from raw milk to AMC, which can provide an effective reference and help in the clinical treatment of *E.coil* infections.

## 2. Results

### 2.1. MIC Results

According to CLSI (2024), MIC value breakpoints of *E. coli* against AMC are presented in Table 1. It showed that out of 43 *E. coli* strains, the susceptibility rate to AMC was 88.37%, intermediary rate was 9.30% and resistance rate was 2.33%.

### 2.2. K-B Results

**Figure 1:**
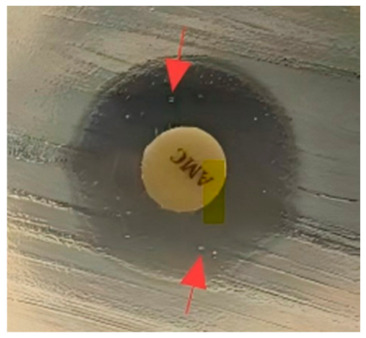
Schematic representation of heteroresistance to amoxicillin/clavulanic acid.

Based on the antimicrobial susceptibility results, isolates classified as “S” and “I” to AMC were selected for preliminary heteroresistance screening. All 15 selected isolates exhibited scattered colonies within the AMC inhibition zone and were preliminarily identified as *E. coli* strains with AMC heteroresistance. A representative result of the AMC disk diffusion test is shown in Figure 1.

### 2.3. PAP Results

**Figure 2:**
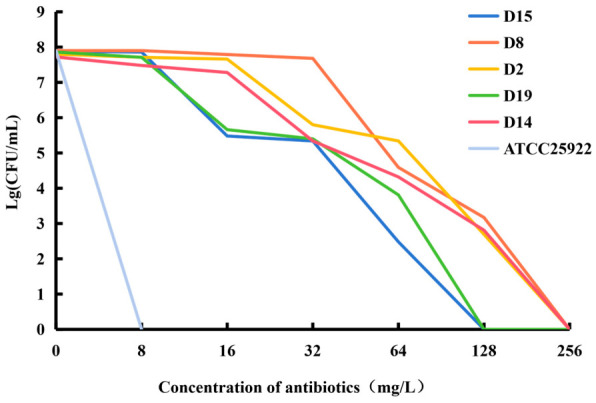
Population analysis profiling curves of *E. coli*.

After confirmation by the PAP assay, 5 of the 15 preliminarily positive isolates met the criteria of MNIC/MIC > 8 and a resistant subpopulation frequency > 1 × 10^−7^, and were finally identified as AMC-heteroresistant *E. coli*. The variation in five *E. coli* strains (D2, D8, D14, D15, and D19) on AMC antibiotics is shown in Figure 2. The figure shows a gradual decrease in resistance to AMC with increasing antibiotic concentration in the range of 0–256 mg/L for the five *E. coli* strains, indicating that they are not resistant to AMC. The highest AMC concentration (highest non-inhibitory concentration) that had no effect on the growth of the dominant populations of strains D2, D8, and D14 was 128 mg/L, and the highest non-inhibitory concentration of D15 and D19 was 64 mg/L. Except for *E. coli* ATCC25922, which did not grow in any of the drug-containing plates, all the other bacteria grew in scattered colonies on the drug-containing plates. According to the criteria for determining heteroresistance, strains D2, D8, D14, D15, and D19 were initially determined to be heteroresistant bacteria, and the frequency of the heteroresistant subpopulations ranged from 3.95 × 10^−6^ to 8.11 × 10^−5^. Two of the five isolates of the heteroresistant subpopulations exhibited multidrug resistance (Table 2).

### 2.4. Results of Resistance Stability

The five heteroresistant subpopulations were passaged for 30 generations under antibiotic-free stress, and the MICs were determined every five generations. The MICs of the first-generation D2-R, D8-R, D14-R, D15-R, and D19-R resistant subpopulations isolates were 32/16, 32/16, 16/8, 4/2, and 4/2 mg/L, respectively (Table 3). The MICs of the D8-R, D14-R, D15-R, and D19-R heteroresistant subpopulations recovered to 2/1 mg/L after five generations of transmission under antibiotic-free conditions, and only D2-R maintained a MIC of 32/16 mg/L. Thus, the resistance of D2-R was more stable. The four heteroresistant subpopulations, D8-R, D14-R, D15-R, and D19-R, were not stable until their MICs returned to the same resistance as the parental strain after 30 generations of transmission.

### 2.5. Fitness Cost

**Figure 3:**
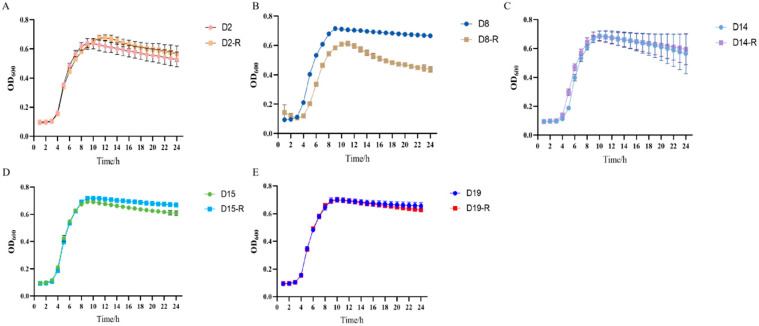
Growth curves of five *E. coli* parental strains and their heteroresistant subpopulation isolates. (**A**) Parental strains of *E*. *coli* D2 and their heteroresistant subpopulation isolates. (**B**) Parental strains of *E*. *coli* D8 and their heteroresistant subpopulation isolates. (**C**) Parental strains of *E*. *coli* D14 and their heteroresistant subpopulation isolates. (**D**) Parental strains of *E*. *coli* D15 and their heteroresistant subpopulation isolates. (**E**) Parental strains of *E*. *coli* D19 and their heteroresistant subpopulation isolates.

The growth curves for all five heteroresistant strains and derived subpopulations displayed the classic pattern of bacterial growth, progressing through lag, exponential, and stationary phases (Figure 3). A two-way ANOVA was performed to comparatively analyze the differences between the D2 parental strains and their heteroresistant subpopulation strains based on the data of their exponential growth periods (*p* > 0.05) (Figure 3A), which indicated that the D2 heteroresistant strains and their heteroresistant subpopulation strains had the same growth rate without growth lag. Similarly all growth curves of D14, D15, D19 and their four heteroresistant subpopulation isolates showed typical bacterial growth curves including lagging, exponential and stabilizing phases, and comparative analysis of differences between them (*p* > 0.05) (Figure 3C–E), which indicated that the D14, D15, D19 heteroresistant strains and their heteroresistant subpopulation strains had the same growth rate without growth lag.

Based on the data of the exponential growth period of D8 heteroresistant strains and its heteroresistant subpopulation strains, a two-way ANOVA was performed to comparatively analyze the differences between them (*p* < 0.01) (Figure 3B), which indicated that there was a growth lag between their heteroresistant subpopulation strains with significantly lower growth rates than that of the D8 heteroresistant strains. Thus, growth of D8 heteroresistant subpopulation isolates caused growth adaptation costs.

### 2.6. Biofilm Formation Results

**Figure 4:**
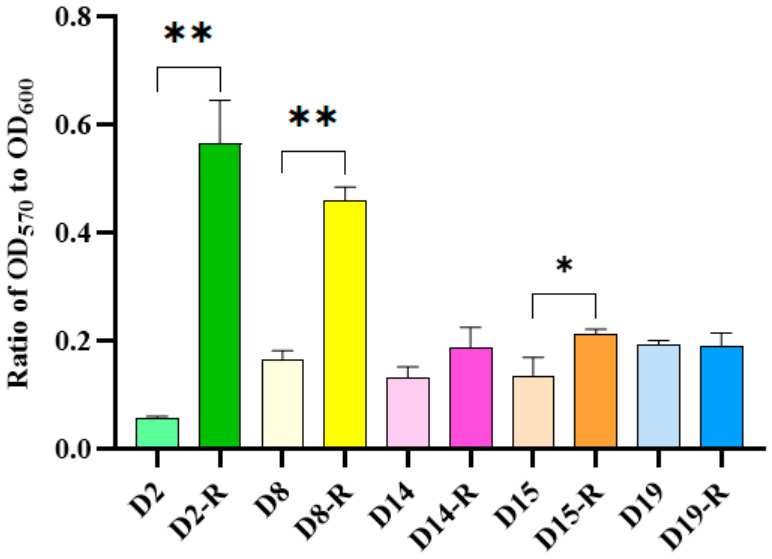
Quantitative analysis of the yield of biofilms. Notes: *, *p* < 0.05; **, *p* < 0.01.

The amount of biofilm production is also one of the main reasons for the development of drug resistance in bacteria; therefore, clarifying whether there is a change in the biofilm production capacity of heteroresistant strains and heteroresistant subpopulations can provide information about drug resistance. The quantitative analysis of the biofilm production of five heteroresistant strains and heteroresistant subpopulations of isolates is shown in Figure 4. The OD_570_ and OD_600_ results represent the biofilm yield and planktonic cell density, respectively. The biofilm yields of heteroresistant subpopulations D2-R and D8-R were highly significantly greater than those of heteroresistant strains, the biofilm yield of heteroresistant subpopulation D15-R was significantly greater than that of heteroresistant strains, and the biofilm yields of heteroresistant subpopulations D14-R and D19-R were similar to those of heteroresistant strains.

### 2.7. Transcriptomic Results

#### 2.7.1. Volcano Plots

**Figure 5:**
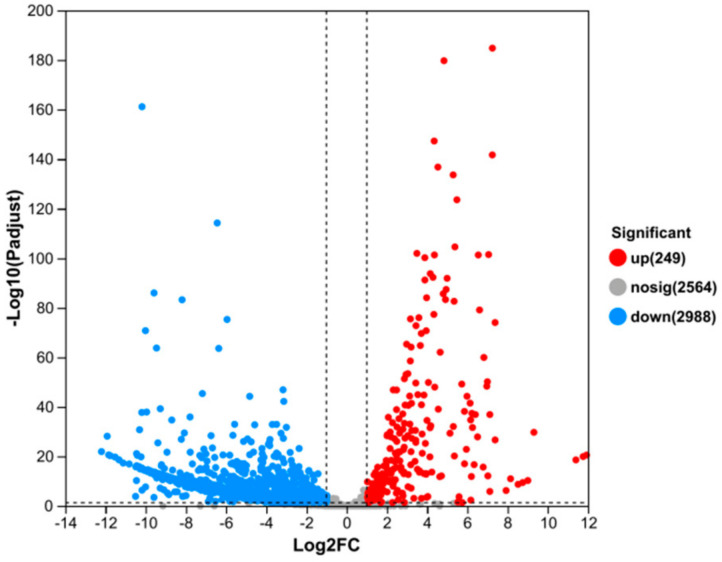
Volcano plot of differentially expressed genes (DEGs) based on RNA-seq for heteroresistant *E. coli* D2 parental strains and their resistant subpopulations. The dots in the diagram represent individual genes, with blue dots indicating down-regulated differentially expressed genes and red dots indicating up-regulated differentially expressed genes. The vertical dashed lines indicate Log_2_FC = ±1 (2-fold expression change cutoff). The *x*-axis and *y*-axis represent log2 of the fold change and t-statistic as-log10 of the value of *p*, respectively. The genes depicted in red (up-regulated) and blue (down-regulated) are differentially expressed genes with >2-fold change and a value of *p* < 0.05.

A total of 5801 genes were detected by *E. coli* D2 RNA-seq analysis, of which 3237 genes were significantly differentially expressed and 2564 genes were not differentially regulated. Figure 5 shows that in the volcano plot of the D2 strain, 249 genes were up-regulated and 2988 genes were down-regulated in their expression levels. Table 4 shows information on the top eight most significantly up-regulated genes in the resistant subpopulations of the heteroresistant strain D2, all derived from known mRNAs. gene1836 (*insB*) encodes the insertion element IS1 protein insB, gene5453 (*ilvC*) encodes the ketol-acid reductoisomerase, gene2462 (*cspA*) encodes the transcription antiterminator/RNA stability regulator CspE, gene4304 (*lacY*) encodes lactose permease, gene4303 (*lacZ*) encodes beta-galactosidase, gene5500 (*atpD*) encodes F0F1 ATP synthase subunit beta, gene5495 (*atpE*) encodes F0F1 ATP synthase subunit C, and gene5498 *(atpA*) encodes F0F1 ATP synthase subunit alpha. The up-regulated expression of genes in the resistant subpopulation of strain D2 is likely to be associated with the development of heteroresistance to AMC in *E. coli*.

#### 2.7.2. GO Enrichment Analysis

**Figure 6:**
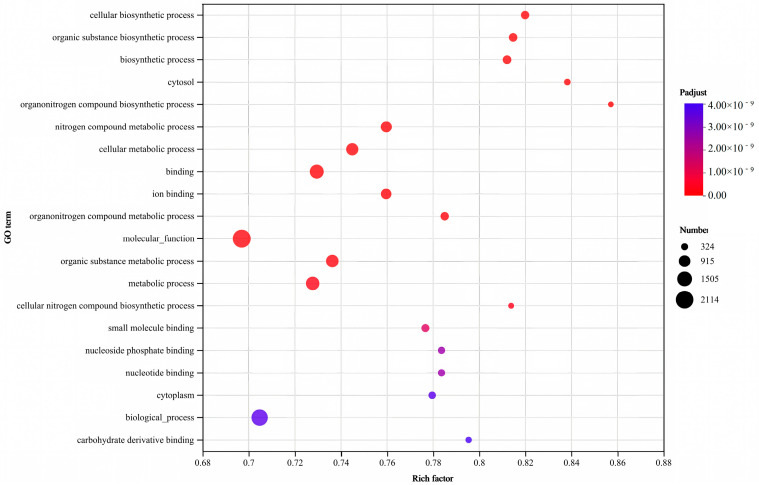
Functional classification of differential gene expression GO terms.

In this study, we revealed the multidimensional functional regulatory network of 2448 significantly differentially expressed genes obtained from the screening of resistant subpopulations of D2 strains by systematic GO functional annotation analysis (Figure 6). The differentially expressed genes were significantly enriched in 33 functional entries in the three major GO categories, including 13 in biological process (BP), 2 in cellular component (CC), and 18 in molecular function (MF).

Biological process genes were mainly enriched in the entries of inter/intraspecific interactions, bioregulation, metabolic processes and stress response. The significant enrichment of group sensing-related genes (*luxR* family transcriptional regulators) suggested that the strains might coordinate the drug resistance phenotype through inter-microbial signaling, while the activation of oxidative stress response pathways (*sodA*, *katE*) might maintain cellular homeostasis by scavenging antimicrobial drug-induced reactive oxygen species. Cellular component genes were significantly enriched in cellular anatomical entities and protein complexes. Notably, the co-enrichment of genes related to outer membrane pore proteins (*ompF*/*ompC*) and multidrug efflux pumps (*AcrAB-tolC*) implied that drug resistance-related structural remodeling might involve the dynamic assembly of cellular membrane components or functional complexes, and that molecular functionally differentiated genes were extensively involved in binding, catalytic activity, translocation and molecular regulation. Among them, the β-lactamase activity-related gene ampD was significantly up-regulated, which directly corroborated the adaptive evolution of the strain to amoxicillin/clavulanic acid, reflecting that the resistance phenotype may depend on multiple molecular mechanisms such as transmembrane transport and regulation of metabolic enzyme activities.

**Figure 7:**
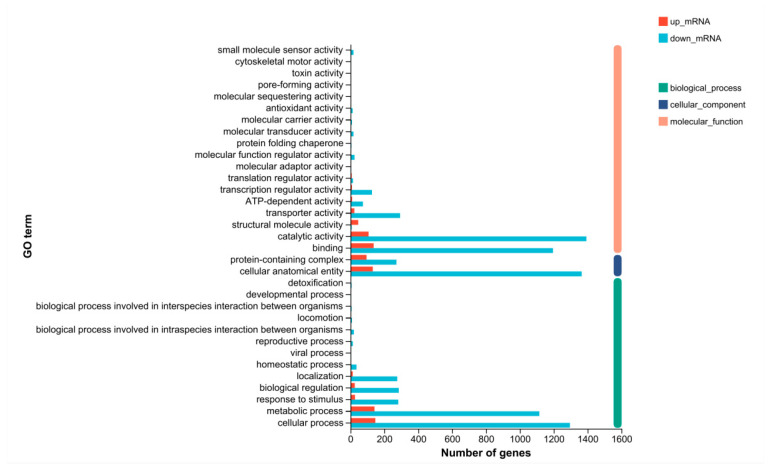
GO term enrichment classification of significantly up- and down-regulated differentially expressed genes in strain D2.

The regulatory features of the differentially expressed genes of the drug-resistant subpopulation were revealed by GO functional enrichment analysis (Figure 7). The 2448 differentially expressed genes screened were significantly enriched only in down-regulated genes in seven entries, such as detoxification function, developmental process, and cytoskeletal motility, suggesting that the drug-resistant strains may reduce energy consumption by inhibiting detoxification metabolism and reducing motility, etc. The rest of the 26 entries (e.g., metabolic process, transporter activity, and toxin activity, etc.) had bi-directional regulation, with the genes related to transporter activity showing a significant up-regulation. Notably, the molecular function category had the highest percentage of genes with catalytic activity and binding function.

#### 2.7.3. KEGG Enrichment Analysis

**Figure 8:**
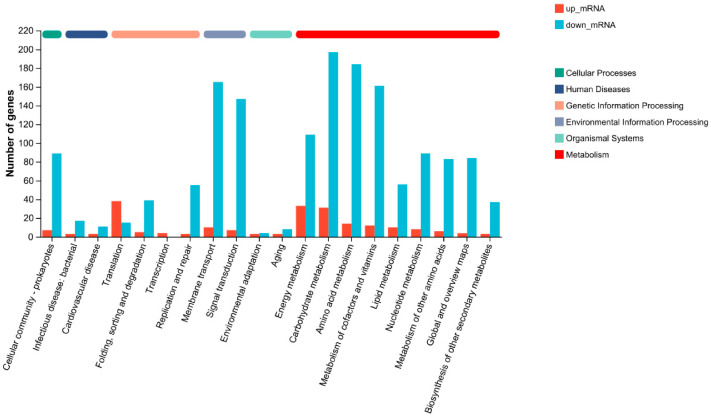
KEGG functions enrichment classification of significantly up-regulated and down-regulated differentially expressed genes in strain D2.

In this study, 2448 significantly differentially expressed genes obtained from RNA-seq screening of the drug-resistant subpopulation of strain D2 were systematically analyzed for KEGG pathway enrichment. As shown in Figure 8, the differential genes showed significant enrichment characteristics in 30 major biological pathways, with metabolism-related pathways having the most enriched entries, while cellular process-related pathways had relatively few. Notably, at the metabolic pathway level, amino acid metabolism pathways gathered the largest proportion of down-regulated genes, while at the genetic information processing level, translation-related pathways showed significant up-regulated gene enrichment.

Focusing on the mechanisms related to antimicrobial resistance, this study identified four key pathways with significant regulatory features (Figure S1): β-lactamase resistance pathway, lipopolysaccharide biosynthesis pathway, *E. coli* biofilm pathway, and bacterial secretion system pathway. Specifically, the β-lactamase resistance pathway (Figure S1A) showed the synergistic effect of multiple resistance mechanisms: the expression of the outer membrane pore protein *ompC/ompF* was significantly down-regulated, suggesting a decrease in the permeability of the cell membrane; at the same time, the expression of the key component of the efflux pumping system, the *AcrAB-tolC* gene cluster, was up-regulated, and the expression of the gene coding for penicillin-binding proteins, *ftsI*, was abnormally high, suggesting that the modification of drug targets might be jointly involved in the formation of drug resistance. The lipopolysaccharide biosynthesis pathway (Figure S1B) showed a typical resistance-associated modification pattern: *eptB*, a lipid A phosphoethanolamine transferase gene, was significantly up-regulated, which may enhance drug resistance by decreasing the permeability of the cationic antimicrobial peptide; on the contrary, down-regulation of the expression of the *pagP* gene may affect the stability of the outer membrane. The biofilm formation pathway (Figure S1C) revealed the activation of biofilm-associated regulatory networks: extracellular matrix synthesis genes such as *Crr*, *fihD*, and *bcsA* were significantly up-regulated, while the expression of the population-sensing regulator *lsrR* was enhanced, suggesting that the enhanced biofilm membrane formation ability may be involved in drug resistance as a physical barrier. The bacterial secretion system pathway (Figure S1D) showed a general up-regulation of the key component genes of the Sec-SRP secretion system (*secD/F*), a phenomenon that may indirectly enhance the drug resistance phenotype by promoting secretion of virulence factors or assembly of efflux pumps.

Notably, the aberrant expression of *ftsI* genes echoes the clinically reported resistance mechanism of penicillin-binding protein mutations, whereas the global up-regulation of genes of the sec system provides new clues for the study of the secretion system-mediated resistance mechanism.

#### 2.7.4. qRT-PCR Results

**Figure 9:**
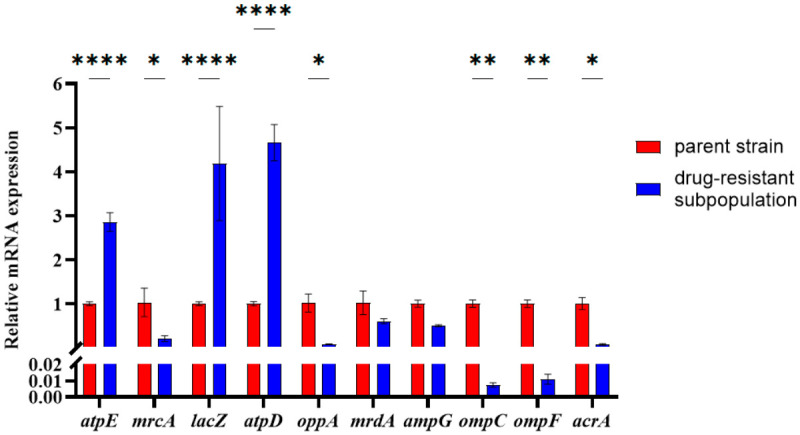
qRT-PCR validation plot of some of the differentially expressed key genes validated by qRT-PCR in strain D2. Notes: *, *p* < 0.05; **, *p* < 0.01; ****, *p* < 0.0001.

The qRT-PCR validation of some key DEGs in the drug-resistant subpopulations of the D2 strain is shown in Figure 9. The up- and down-regulated expression of key genes in the drug-resistant subpopulations of the D2 strain shown in Figure 9 is consistent with the results of the transcriptome analysis, of which three genes were up-regulated, including *atpE* encoding F0F1 ATP synthase subunit C, *lacZ* encoding beta-galactosidase, and *atpD* encoding F0F1 ATP synthase subunit beta. Seven genes were down-regulated, including *mrcA* encoding peptidoglycan glycosyltransferase/peptidoglycan DD-transferase; *oppA* encoding putative transporter ectoplasmic proteins; *mrdA* encoding peptidoglycan DD-transferase; *ampG* encoding the furfuryl peptide MFS transporter; *ompC* and *ompF* encoding pore proteins; and acrA encoding the multidrug efflux RND transporter ectoplasmic adaptor subunit. All identified DEGs showed a transcriptional fold change higher than 2-fold in the resistant subpopulations of D2 strains.

## 3. Discussion

Currently, antibiotics remain the first-line choice for the clinical treatment of *E. coli* infections [31]. However, when encountering heteroresistant *E. coli*, the resistant subpopulations within the bacterial population cannot be completely eradicated, leading to treatment failure, persistent and recurrent infections. This poses challenges for effective clinical prevention and control and impacts the economic benefits and healthy development of the livestock industry [32]. Therefore, in-depth research into the heteroresistance of milk-derived *E. coli* is particularly important.

In this study, the susceptibility of 43 isolated *E. coli* strains to amoxicillin/clavulanic acid (AMC) was tested using the micro-broth dilution method. The results showed sensitivity, intermediate, and resistance rates of 88.37%, 9.30%, and 2.33%, respectively. The disk diffusion test performed on sensitive and intermediate strains revealed that 15 strains exhibited scattered colonies within the AMC inhibition zone, preliminarily identified as having heteroresistance to AMC. Further confirmation using the population analysis profile (PAP) method identified strains D2, D8, D14, D15, and D19 as heteroresistant, with resistant subpopulation frequencies ranging from 3.95 × 10^−6^ to 8.11 × 10^−5^. A study by Liao et al. [33] reported heteroresistant subpopulation frequencies for *E. coli* against colistin ranging from 4.0 × 10^−7^ to 4.0 × 10^−6^. Compared to this, the frequency of heteroresistance to AMC observed in our study is relatively high, suggesting specific selection under the pressure of this particular drug.

### 3.1. Stability and Fitness Costs of Heteroresistant Subpopulations

Based on their formation mechanisms, heteroresistant bacteria can be divided into stable and unstable resistant subpopulations [34]. This study found that most resistant subpopulations (D8-R, D14-R, D15-R, D19-R) were unstable; their MIC values rapidly reverted to the level of the parental strain after serial passage in antibiotic-free medium. Only the resistance of D2-R remained stable (MIC maintained at 32/16 mg/L) after 30 passages. This result aligns with the report by Kuang et al. [35], who found that only 1 out of 17 resistant subpopulations were stable. Research by Band et al. [36] also demonstrated that resistant subpopulations could regain susceptibility after passage in drug-free medium. This suggests that the resistance of most heteroresistant subpopulations may not reach a stable, clinically relevant level. However, under sustained antibiotic selection pressure, these subpopulations have the potential to evolve and acquire resistance at or above clinical breakpoints, thereby becoming clinically resistant.

Analysis of fitness costs revealed that, except for the D8 resistant subpopulation, which showed a significantly lower growth rate than its parental strain (*p* < 0.01) indicating a growth fitness cost, the growth curves of the resistant subpopulations of the other four strains (D2, D14, D15, D19) showed no significant difference from their parental strains (*p* > 0.05), with no observed growth lag. This indicates that some heteroresistant subpopulations (e.g., D2-R) can maintain a stable resistant phenotype without incurring significant growth costs, potentially enhancing their persistence in the infection environment.

### 3.2. Enhanced Biofilm Formation as an Important Phenotype of Heteroresistance

Bacterial biofilms are complex structures composed of microbial communities and their self-secreted extracellular substances. They represent a crucial mechanism for *E. coli* to enhance tolerance to the host immune system and antimicrobial agents, and are a significant contributor to bacterial drug resistance [37]. Processes within the biofilm, such as spontaneous mutations, external stimuli, internal microenvironments, and interspecies interactions, can lead to the formation of widely divergent subpopulations within the biofilm layer. A higher biofilm biomass is often associated with more pronounced heterogeneity [24,25]. This study found that the biofilm formation capacity of resistant subpopulations D2-R and D8-R was highly significantly greater than that of their parental strains, that of D15-R was significantly greater, while those of D14-R and D19-R were similar to their parental strains. This finding is consistent with Mezcord et al. [38], who confirmed that derivatives of AMA40 IHC1 and IHC2 also exhibited increased biofilm formation. The increase in biofilm production may not only act as a physical barrier limiting antibiotic penetration but the heterogeneous microenvironment within the biofilm may also directly promote the formation and selection of resistant subpopulations [39]. Therefore, enhanced biofilm formation capacity is a significant phenotypic characteristic associated with the development of heteroresistance.

### 3.3. Down-Regulation of Outer Membrane Porin Expression as a Key Molecular Mechanism of Heteroresistance

To investigate the molecular basis of heteroresistance, transcriptomic sequencing was performed on the typical heteroresistant strain D2 and its resistant subpopulation. A total of 3237 genes showed significant differential expression, with 249 up-regulated and 2988 down-regulated. KEGG pathway enrichment analysis revealed key pathways associated with AMC resistance, with the beta-lactam resistance pathway being particularly prominent.

In-depth analysis of this pathway showed significant down-regulation in the expression of the genes encoding the outer membrane porins *ompC* and *ompF* in the resistant subpopulation. *OmpC* and *ompF* are major outer membrane porins of *E. coli* and serve as important channels for the transmembrane transport of various drugs, including β-lactams [40]. Reduced expression of these porins is closely associated with decreased susceptibility/increased resistance to β-lactam antibiotics [41,42,43]. Concurrently, the expression of *envZ*, encoding the sensor histidine kinase of the two-component system that regulates these porins, was also down-regulated. *EnvZ* senses environmental osmotic changes and regulates the phosphorylation state of the transcription factor *OmpR*, which in turn controls the transcription of *ompF* and *ompC* [44]. Down-regulation of *envZ* leads to reduced or absent expression of the *ompC* and *ompF* porins, thereby preventing antibiotics from crossing the outer membrane and entering the cell, resulting in bacterial resistance. Therefore, the reduced or absent expression of the *ompF* and *ompC* porins is a key molecular mechanism contributing to the heteroresistance of *E. coli* to AMC.

### 3.4. Synergistic Effects of Other Related Molecular Pathways

Transcriptomic analysis also revealed that the development of heteroresistance involves that of other pathways. In the lipopolysaccharide biosynthesis pathway, the lipid A phosphoethanolamine transferase gene *eptB* was significantly up-regulated, which may enhance resistance by reducing the permeability of the outer membrane to cationic antimicrobial peptides. In the bacterial secretion system pathway, key component genes of the Sec-SRP secretion system (*secD/F*) were generally up-regulated. This may indirectly contribute to the resistant phenotype by promoting the secretion of virulence factors or the assembly of efflux pumps. These coordinated changes across multiple pathways and genes collectively form the complex resistance network of the resistant subpopulation.

### 3.5. Validation of Key Differentially Expressed Genes

qRT-PCR validation of 10 key differentially expressed genes yielded results highly consistent with the transcriptomic analysis. Genes associated with biofilm formation and stress adaptation (e.g., *atpE*, *atpD*, *lacZ*) were up-regulated, while genes related to cell wall synthesis, substance transport, and outer membrane porins (e.g., *mrcA*, *oppA*, *mrdA*, *ampG*, *ompC*, *ompF*, *acrA*) were down-regulated. This further confirms, at the molecular level, the multiple adaptive adjustments of the heteroresistant subpopulation in metabolism, barrier function, and stress response.

In conclusion, the resistant subpopulation of the typical heteroresistance strain D2 exhibited stable resistance without growth lag. Its resistance mechanism is multifaceted: First, the down-regulation of outer membrane porins *ompF* and *ompC* expression is the core mechanism limiting antibiotic uptake. Second, a significant enhancement in biofilm formation capacity provides physical protection and a heterogeneous microenvironment. Third, alterations in other molecular pathways, such as lipopolysaccharide modification and secretion system activation, also contribute to the resistant phenotype. These findings indicate that heteroresistance is not caused by a single mechanism but is the result of the combined action of multiple biological processes. Through systematic characterization and mechanistic analysis, this study provides new insights and a theoretical basis for a deeper understanding of the complex network underlying heteroresistance in *E. coli*.

## 4. Materials and Methods

### 4.1. Strains, Media, and Reagents

Standard strain: *E.coli* ATCC29522 was purchased from ATCC (Manassas, VA, USA); experimental strains: The 43 *E. coli* isolates used in this study were all isolated from 84 raw milk samples collected between 2022 and 2023 from a dairy farm in Hutubi, Xinjiang, China. These strains were isolated and preserved in −80 °C glycerol stocks by the Xinjiang Academy of Agricultural Sciences. MacConkey agar medium, Mueller–Hinton agar (MHA), LB broth, and 0.85% normal saline were purchased from Beijing Road and Bridge Technology Co., Ltd. (Beijing, China). Antimicrobial susceptibility testing plates and Kirby–Bauer (K-B) paper disks were obtained from YINOCON (Tianjin) Biotechnology Co., Ltd. (Tianjin, China). The TRNzol Universal Total RNA Extraction Kit, FastKing cDNA First Strand Synthesis Kit, FastReal Rapid Fluorescent Quantitative PCR Kit, and Bacterial DNA Extraction Kit were all sourced from Tiangen Biochemical Technology Co., Ltd. (Beijing, China). Additionally, DNA markers, agarose, nucleic acid dyes, 50× TAE buffer, and 2× Taq PCR MasterMix were procured from Xinjiang Dingfeng Biotechnology Co., Ltd. (Urumqi, Xinjiang, China).

### 4.2. Drug Susceptibility Testing

Forty-three strains were tested for susceptibility to AMC antimicrobial drugs according to the broth dilution method (CLSI, 2024) recommended by the American Committee for Clinical and Laboratory Standardization. The bacterial solutions were adjusted to 0.5 McFarland turbidity using a McFarland turbidimeter and inoculated into 96-well plates containing different concentrations of AMC to determine the MIC of AMC. The drug-sensitive plates were placed in an incubator at 35 °C for 18–20 h. Where bacterial growth was present in small wells with diffuse turbidity or rounded precipitation, the lowest concentration of AMC contained in wells without bacterial growth was the MIC, and the blank control wells should have been free of bacterial growth. According to CLSI (2024) [45], the MIC values of *E. coli* against AMC were as follows: susceptible strains (≤8/4 mg/L), intermediate strains (16/8 mg/L) and resistant strains (≥32/16 mg/L).

### 4.3. Kirby–Bauer Test

Fresh colonies were picked with a sterile inoculating ring and inoculated into 0.85% saline, shaken well, and made into a bacterial suspension with 0.5 McFarland turbidity (1 × 10^8^ CFU/mL). A total of 100 μL of the bacterial suspension was sucked and inoculated on the MHA plate, and then the surface of the medium was spread uniformly with the “L” type spreading stick until the surface of the medium was completely dry and the antimicrobial susceptibility testing disks (30 μg, AMC) were pasted onto the center of the plate with sterile tweezers. The plate with the drug-sensitive paper was placed in the incubator, the temperature was controlled to be 37 °C ± 1 °C, and the plate was incubated for 18–24 h to observe whether there was any bacterial growth in the ring of inhibition, and the results were interpreted with reference to CLSI (2024).

### 4.4. Population Analysis Profile

Using the PAP method [15], MHA plates with antibiotic concentrations of 0–256 mg/L were sequentially prepared. Fresh colonies were inoculated in 0.85% saline, shaken well, and made into a suspension of 0.5 mM turbidity (1 × 10^8^ CFU/mL), and then gradient dilution was carried out to make suspensions of 10^3^, 10^4^, 10^5^, 10^6^, 10^7^, and 10^8^, respectively. The suspensions of different gradients were inoculated onto MHA plates with different antibiotic concentrations and spread evenly with an “L” shaped spreading bar, with two parallels for each gradient. The inoculated and evenly coated MHA plates were placed upside down in an incubator at 37 °C ± 1° C and incubated for 24 h. The antibiotic plates were removed from the incubator, and the colonies grown on each plate were counted, then converted to the actual number of colonies grown according to the previous dilution, and plotted with the antibiotic concentration (mg/L) as the horizontal coordinate and the Lg (CFU/mL) as the vertical coordinate. If the ratio of MNIC to MIC was greater than 8 and the frequency of the resistant subpopulation was greater than 1 × 10^−7^, then it could be recognized as an heteroresistance strain [14,15]. The control strain was *E. coli* ATCC25922.

### 4.5. Resistance Stability Determination

Bacteria growing on the plate with the highest antimicrobial concentration were picked as the heteroresistant subpopulation, which was recorded as strain-R. The MIC value was determined once every five generations on MHA plates without antimicrobial concentration for 30 passages. The change in the stability of the resistant subpopulation was observed; if it still maintained the resistant phenotype, it was a stable heterogeneous resistant strain, and if it recovers its sensitivity, it was an unstable heterogeneous resistant strain.

### 4.6. Fitness Cost Determination

Single colonies of heteroresistant strains and their subpopulations were inoculated into LB liquid medium and incubated at 37 °C, 180 r/min overnight. The bacterial suspension was prepared at a concentration of 1 × 10^7^ CFU/mL, and 10 μL was added into a 96-well plate containing 100 μL LB broth per well, incubated at 37 °C and 180 r/min on a shaker, and the OD_600_ value was measured every 1 h for 24 h. The growth curve was plotted according to the OD_600_ value, and the growth rate was compared. The entire procedure was repeated three times.

### 4.7. Biofilm Formation Assay

Heteroresistance parental strains and subpopulations were taken from the overnight culture, diluted 50 times, and inoculated into glass test tubes containing LB broth. LB broth was used as a negative control, and three parallel experiments were set up, and incubated at 37 °C for 24 h. OD_600_ was measured by using an enzyme labeler to determine the total biomass. The samples were washed three times with PBS buffer, inverted to dry, stained with crystalline ammonium violet oxalate for 15 min, rinsed with monodistilled water and dried; 150 μL of 95% alcohol was added to each well, the reaction was performed at room temperature for 30 min and the OD_570_ of each sample was measured. Biofilm formation was determined as the OD_570_/OD_600_ ratio as a test for the minimization of the growth difference between samples.

### 4.8. Transcriptomics Analysis

Heteroresistance parental strains and resistant subpopulations cultured to logarithmic growth stage were inoculated in LB medium and incubated in a thermostatic shaker at 37 °C and 180 rpm for 5 h. Then the organisms were collected by centrifugation at 4 °C and 15,000 rpm/min for 5 min, and the supernatant was discarded to keep the precipitated organisms, which were washed by adding sterile water 1–2 times, and then transferred to centrifuge tubes. Transcriptomic sequencing was performed by Meiji Biotechnology Co. (Shanghai, China). To evaluate the phenotype variation, the Kyoto Encyclopedia of Genes and Genomes (KEGG) and Gene Ontology (GO) enrichment analyses of significant differentially expressed genes (DEGs) were performed based on a hypergeometric test. Significance levels were corrected with a rigorous threshold of q ≤ 0.05.

### 4.9. Quantitative Real-Time Polymerase Chain Reaction Validation

In transcriptomic analysis, 10 significant DEGs of D2 strain were selected for quantitative real-time polymerase chain reaction (qRT-PCR) validation, including three expression up-regulated genes: gene5495 (*atpE*), gene5495 (*lacZ*), and gene5500 (*atpD*); and seven expression down-regulated genes: gene0359 (*mrcA*), gene3050 (*oppA*), gene4001 (*mrdA*), gene4211 (*ampG*), gene2006 (*ompC*), gene3673 (*ompF*), and gene4176 (*acrA*). Total RNA extraction, PCR primer design and synthesis, and experimental procedures [46] are reported. Relative gene expression levels were calculated using the 2^−ΔΔCt^ method, with the 16S rRNA gene used as the endogenous reference for normalization. Each sample was assayed in triplicate. Gene expression differences were expressed as fold change. Primer sequences are listed in the Supplementary Table S1.

### 4.10. Statistical Analysis

Two independent experiments were conducted for each test in triplicate. All data were recorded and organized in Microsoft Excel 2019 for statistical analysis. Statistical analyses between groups were performed using independent *t*-tests, with *p* < 0.01 indicating a highly significant difference and *p* < 0.05 indicating a significant difference.

## 5. Conclusions

*E.coli* strains D2, D8, D14, D15, and D19 were heteroresistant to AMC, and the frequency of heteroresistant subpopulations ranged from 3.95 × 10^−6^ to 8.11 × 10^−5^. The resistant subpopulation of typical heteroresistant strains of D2 were stable and did not have a growth lag. The mechanism of heteroresistance produced by resistant subpopulations of different strains may be different, and their heteroresistance is often not produced by a single mechanism. The mechanism of heteroresistance in the typical heteroresistant strain D2 involves *ompF*, reduced or absent *ompC* pore protein expression, and increased formation of biofilm.

## Figures and Tables

**Table 1:** Results of the MIC test of AMC against 43 strains of *E. coli*.

Antibacterial Drug Name	Type of Drug Sensitivity	MIC Folding Point/(mg/L)	Number of Strains	Ratio/%
Amoxicillin/ Clavulanic acid	S	≤8/4	38	88.37%
	I	16/8	4	9.30%
	R	≥32/16	1	2.33%

Notes: S, Sensitive; I, Intermediary; R, Resistant.

**Table 2:** Susceptibility characteristics of the five *E. coli* isolates studied.

Isolate	Broth MIC of ACM (mg/L)	Highest Concentration of Growth in PAPs (mg/L)	Proportion of Resistant Subpopulations	Resistant Colonies MIC After 30 Generations of Daily Passages on Antibiotic-Free Media	Population Analysis Profiling	The MICs of Clinical Routine Antimicrobial Agents (mg/L) ^a^							
						AMP	CEP	GEN	TET	FLO	CIP	S	SIZ
D2	1/0.5	128	7.94 × 10^−6^	32/16	Hetero- resistant	128	>128	0.5	0.25	64	0.25	4	8
D8	1/0.5	128	6.25 × 10^−6^	2/1	Hetero- resistant	64	>128	0.5	0.5	64	0.25	2	8
D14	2/1	128	1.13 × 10^−5^	2/1	Hetero- resistant	4	32	0.5	1	4	0.25	4	16
D15	2/1	64	3.95 × 10^−6^	2/1	Hetero- resistant	2	4	0.25	1	4	0.25	2	256
D19	1/0.5	64	8.11 × 10^−5^	2/1	Hetero- resistant	4	2	0.25	2	4	0.25	2	64

Notes: Broth MIC of AMC represents the MIC of AMC for the parental strain determined by broth microdilution. Resistant Colonies MIC represents the MIC of AMC for the resistant subpopulation after 30 consecutive daily passages on antibiotic-free medium, which was used to evaluate the genetic stability of the resistant phenotype. ^a^ For heteroresistant isolates, the objects of MICs detection are resistant subpopulations selected from PAPs. Abbreviations: AMP, Ampicillin; CEP, cephalothin; GEN, Gentamicin; TET, Tetracycline; FLO, Florfenicol; CIP, Ciprofloxacin; S, streptomycin; SIZ, Sulfafurazole.

**Table 3:** MIC changes in AMC-heteroresistant subpopulations during 30 consecutive daily passages on antibiotic-free medium (determined at 5-generation intervals).

Generation of Passages	D2-R (mg/L)	D8-R (mg/L)	D14-R (mg/L)	D15-R (mg/L)	D19-R (mg/L)
1 generation	32/16	32/16	16/8	4/2	4/2
5 generations	32/16	16/8	16/8	4/2	2/1
10 generations	32/16	16/8	4/2	2/1	2/1
15 generations	32/16	4/2	4/2	2/1	2/1
20 generations	32/16	4/2	2/1	2/1	2/1
25 generations	32/16	2/1	2/1	2/1	2/1
30 generations	32/16	2/1	2/1	2/1	2/1

**Table 4:** The eight most significantly up-regulated genes in strain D2.

Gene_Id	Gene Name	Gene Description	Log_2_FC	Padjust
gene1836	*insB*	insertion element IS1 protein insB	11.908867	2.77 × 10^−12^
gene5453	*ilvC*	ketol-acid reductoisomerase	7.2274730	1.25 × 10^−185^
gene2462	*cspA*	transcription antiterminator/RNA stability regulator CspE	7.2232289	1.65 × 10^−142^
gene4304	*lacY*	lactose permease	6.5871662	7.01 × 10^−80^
gene4303	*lacZ*	beta-galactosidase	6.5263219	4.27 × 10^−102^
gene5500	*atpD*	F0F1 ATP synthase subunit beta	5.4605227	2.18 × 10^−124^
gene5495	*atpE*	F0F1 ATP synthase subunit C	5.3231822	2.12 × 10^−83^
gene5498	*atpA*	F0F1 ATP synthase subunit alpha	4.9741557	1.14 × 10^−92^

## Data Availability

The original contributions presented in this study are included in the article/Supplementary Material. Further inquiries can be directed to the corresponding author.

## Supplementary Materials

The following supporting information can be downloaded at https://www.mdpi.com/article/10.3390/antibiotics15070705/s1. Figure S1: KEGG functional pathway diagram of significantly up-regulated and down-regulated differentially expressed genes; Table S1: qRT-PCR primer sequences. Table S2: Significantly differentially expressed genes (DEGs) identified by RNA-seq in the resistant subpopulation of *E. coli* D2.
