# Supplementary material for: Biofilm Formation and the Pore Proteins ompF and ompC Lead to Heteroresistance in Escherichia coli
Source: Antibiotics (Basel). 2026 Jul 20;15(7):705. doi: 10.3390/antibiotics15070705 (PMC13404454; doi:10.3390/antibiotics15070705)
Supplement: Supplementary file 1 [file antibiotics-15-00705-s001.zip › antibiotics-4434448-supplementary.pdf]

Supplementary Materials

Table S1. qRT-PCR primer sequences.

Gene Name	Primer Sequence	Target Fragment (bp)
16S rRNA	GTGTAGCGGTGAAATGCG<br>CGTTTACGGCGTGGACTA	136
atpE	TGGGTCTGGCGGCAATCG<br>CAGCAGAGGAATCAGATCAGGTTG	98
MrcA	AACAAGATTACCGATACGCTGAAGG<br>TCGCCGTCGCTTGCTGAG	94
lacZ	CAGCCGCTACAGCCAACAAC<br>AGGAGTCGTCGCCACCAATC	119
atpD	AGGCGAGATCGGTGAAGAAGAG<br>TTTCCAGCAGTTCCTGAGAGTTTG	89
oppA	AACGAACCAACTTCCTTCCTGAAC<br>GTTTCCGCCATAATGCTGTCAAAG	98
mrdA	GTCTGAAAGCGAACGAAACCTATAATG<br>CGACAGCCACTTGCGGATTG	108
AmpG	GCCGCCAGACGCTTGAATATAC<br>CCGCCGCCAGTGTCCAC	99
ompC	GGCGTTGGCGGTTCTATCAC<br>GCAGCGGTGTTCTGAGCATC	95
ompF	TTGCTACCTATCGTAACTCCAACTTC<br>CAACACCGTCGCCGTTAGAAC	117
acrA	GGCGAATGCTGCGGTAACTG<br>TCGGAGAGGTGACTTTGGTGTAAG	83

Table S2. Significant differentially expressed genes (DEGs) identified by RNA-seq in the resistant subpopulation of *E. coli* D2.

Gene_Id	Gene Name	Gene Description	Log2FC	Padjust
gene5453	ilvC	MULTISPECIES: ketol-acid reductoisomerase	7.227473047	$1.25531588775 \times 10^{-185}$
gene0421	tuf	MULTISPECIES: elongation factor Tu	4.331779442	$4.04818128975 \times 10^{-148}$
gene2462	cspA	MULTISPECIES: transcription antiterminator/RNA stability regulator CspE	7.223228929	$1.65320910114 \times 10^{-142}$
gene2516	gapA	glyceraldehyde-3-phosphate dehydrogenase	4.516903164	$1.33988637681 \times 10^{-137}$
gene5500	atpD	MULTISPECIES: F0F1 ATP synthase subunit beta	5.460522706	$2.18915609 \times 10^{-124}$
gene5350	typA	MULTISPECIES: ribosome-dependent GTPase TypA	3.471847526	$9.65920020001 \times 10^{-103}$
gene2626	lpp	MULTISPECIES: murein lipoprotein Lpp	4.3429608	$4.07911710464 \times 10^{-102}$
gene4303	lacZ	beta-galactosidase	6.526321976	$4.27176061701 \times 10^{-102}$
gene5221	rplJ	MULTISPECIES: 50S ribosomal protein L10	3.866493844	$5.29851178043 \times 10^{-101}$
gene0452	rplQ	MULTISPECIES: 50S ribosomal protein L17	4.137813306	1.51245547037

				$\times 10^{-94}$
gene5324	fdoI	MULTISPECIES: formate dehydrogenase cytochrome b556 subunit	4.272909093	$4.66207816163 \times 10^{-93}$
gene5498	atpA	MULTISPECIES: F0F1 ATP synthase subunit alpha	4.974155775	$1.14349523965 \times 10^{-92}$
gene0420	fusA	MULTISPECIES: elongation factor G	3.868210409	$5.44788631393 \times 10^{-92}$
gene5501	atpC	MULTISPECIES: F0F1 ATP synthase subunit epsilon	4.92517531	$3.61993548822 \times 10^{-88}$
gene5499	atpG	MULTISPECIES: F0F1 ATP synthase subunit gamma	4.780798928	$1.98569936529 \times 10^{-86}$
gene0451	rpoA	MULTISPECIES: DNA-directed RNA polymerase subunit alpha	3.953593978	$8.00974352837 \times 10^{-85}$
gene5495	atpE	MULTISPECIES: F0F1 ATP synthase subunit C	5.323182278	$2.12962269715 \times 10^{-83}$
gene4304	lacY	MULTISPECIES: lactose permease	6.587166241	$7.01172623589 \times 10^{-80}$
gene5218	rpoC	DNA-directed RNA polymerase subunit beta′	4.315380575	$4.31621557671 \times 10^{-78}$
gene0450	rpsD	MULTISPECIES: 30S ribosomal protein S4	3.57055933	$9.11578561092 \times 10^{-77}$
gene0438	rplE	MULTISPECIES: 50S ribosomal protein L5	3.151378473	$2.87021448774 \times 10^{-76}$
gene4213	cyoB	MULTISPECIES: cytochrome o ubiquinol oxidase subunit I	3.426895939	$1.52134987251 \times 10^{-73}$
gene5219	rpoB	DNA-directed RNA polymerase beta chain	3.934552807	$1.68942084398 \times 10^{-71}$
gene1278	eno	MULTISPECIES: phosphopyruvate hydratase	3.691342789	$2.09882620222 \times 10^{-70}$
gene0441	rplF	MULTISPECIES: 50S ribosomal protein L6	2.960018773	$4.5985458603 \times 10^{-66}$
gene0524	rplM	MULTISPECIES: 50S ribosomal protein L13	3.644397977	$1.59832917479 \times 10^{-65}$
gene3692	rpsA	MULTISPECIES: 30S ribosomal protein S1	3.183848038	$6.88214800972 \times 10^{-65}$
gene3904	sucC	MULTISPECIES: ADP-forming succinate--CoA ligase subunit beta	4.630292871	$7.98795734682 \times 10^{-63}$
gene0442	rplR	MULTISPECIES: 50S ribosomal protein L18	3.145294046	$2.78065313219 \times 10^{-59}$
gene5220	rplL	MULTISPECIES: 50S ribosomal protein L7/L12	3.023589892	$3.7371438267 \times 10^{-54}$
gene1709	ptsH	MULTISPECIES: phosphocarrier protein Hpr	2.933039387	$7.4789571847 \times 10^{-54}$
gene1707	ptsI	phosphoenolpyruvate-protein phosphotransferase	2.848501496	$4.25663130629 \times 10^{-52}$
gene0525	rpsI	MULTISPECIES: 30S ribosomal protein S9	4.043839929	$1.31801977353 \times 10^{-50}$
gene4214	cyoC	MULTISPECIES: cytochrome o ubiquinol oxidase subunit III	3.416571331	$2.23155062994 \times 10^{-50}$
gene0703	rpoD	RNA polymerase sigma factor RpoD	2.29711722	$1.33476984958 \times 10^{-47}$

gene4577	rpsB	MULTISPECIES: 30S ribosomal protein S2	2.460773794	$1.4319156318\times 10^{-47}$
gene3381	rpmF	MULTISPECIES: 50S ribosomal protein L32	3.51910594	$1.08551079514\times 10^{-45}$
gene5205	hupA	MULTISPECIES: DNA-binding protein HU-alpha	3.821821406	$1.60547898231\times 10^{-45}$
gene5226	tuf	MULTISPECIES: elongation factor Tu	3.112671329	$4.01469185922\times 10^{-45}$
gene0449	rpsK	MULTISPECIES: 30S ribosomal protein S11	3.20442067	$3.52086643976\times 10^{-42}$
gene1941	nuoCD	MULTISPECIES: NADH-quinone oxidoreductase subunit C/D	3.699449695	$1.35866027683\times 10^{-41}$
gene0419	rpsG	MULTISPECIES: 30S ribosomal protein S7	2.88631024	$1.72967014654\times 10^{-41}$
gene0439	rpsN	MULTISPECIES: 30S ribosomal protein S14	3.048832041	$2.29398700414\times 10^{-41}$
gene3908	sdhA	succinate dehydrogenase flavoprotein subunit	4.535203232	$8.04922033036\times 10^{-40}$
gene4900	valS	valyl-tRNA synthetase	2.460125161	$1.20409795674\times 10^{-39}$
gene0443	rpsE	MULTISPECIES: 30S ribosomal protein S5	2.770292259	$6.45819866304\times 10^{-38}$
gene0437	rplX	MULTISPECIES: 50S ribosomal protein L24	2.050877308	$1.48857117355\times 10^{-36}$
gene0418	rpsL	MULTISPECIES: 30S ribosomal protein S12	2.577376113	$5.74168493238\times 10^{-36}$
gene3906	sucA	2-oxoglutarate dehydrogenase E1 component	3.974919668	$2.78675568612\times 10^{-35}$
gene1441	rplS	MULTISPECIES: 50S ribosomal protein L19	2.257063786	$2.24755882345\times 10^{-34}$
gene1006	pgk	MULTISPECIES: phosphoglycerate kinase	2.849353909	$4.0848857377\times 10^{-34}$
gene5532	rnpA	MULTISPECIES: ribonuclease P protein component	3.119947768	$8.35964762323\times 10^{-34}$
gene0440	rpsH	MULTISPECIES: 30S ribosomal protein S8	2.481315547	$1.25051263526\times 10^{-32}$
gene4520	dnaQ	DNA polymerase III, epsilon subunit	4.127921498	$2.64867035687\times 10^{-32}$
gene5222	rplA	ribosomal protein L1	2.779024023	$9.70779261198\times 10^{-32}$
gene1950	nuoM	NADH-quinone oxidoreductase subunit M	3.397866406	2.98795067875E-31
gene0433	rplP	MULTISPECIES: 50S ribosomal protein L16	3.217069004	$3.36740267635\times 10^{-31}$
gene0401	crp	MULTISPECIES: cAMP-activated global transcriptional regulator CRP	2.144305502	$1.32732119961\times 10^{-30}$
gene1946	nuoI	NADH-quinone oxidoreductase subunit NuoI	3.728034471	$8.75284225677\times 10^{-30}$
gene0431	rplV	MULTISPECIES: 50S ribosomal protein L22	3.014945458	$3.95354210806\times 10^{-29}$
gene1007	fbaA	MULTISPECIES: class II fructose-bisphosphate	2.172936795	1.15532500901

		aldolase		$\times 10^{-28}$
gene0435	rpsQ	MULTISPECIES: 30S ribosomal protein S17	2.880009161	3.97744737531 $\times 10^{-28}$
gene0446	secY	MULTISPECIES: preprotein translocase subunit SecY	2.356867803	6.61073836794 $\times 10^{-28}$
gene0430	rpsS	MULTISPECIES: 30S ribosomal protein S19	3.414081282	2.56192607192 $\times 10^{-27}$
gene1440	trmD	MULTISPECIES: tRNA (guanosine(37)-N1)-methyltransferase TrmD	2.471242102	8.26679566651 $\times 10^{-27}$
gene0429	rplB	ribosomal protein L2	3.417956932	2.08525544919 $\times 10^{-26}$
gene3903	sucD	MULTISPECIES: succinate--CoA ligase subunit alpha	3.893196051	5.30960947389 $\times 10^{-26}$
gene4764	ettA	MULTISPECIES: energy-dependent translational throttle protein EttA	2.137741649	6.35731800862 $\times 10^{-29}$
gene1949	nuoL	MULTISPECIES: NADH-quinone oxidoreductase subunit L	2.798412697	1.8584412014 $\times 10^{-23}$
gene0428	rplW	50S ribosomal protein L23	3.178525013	1.18349561713 $\times 10^{-22}$
gene0062	rpoZ	MULTISPECIES: DNA-directed RNA polymerase subunit omega	2.626660178	1.37804285888 $\times 10^{-22}$
gene0432	rpsC	MULTISPECIES: 30S ribosomal protein S3	2.551668126	1.37804285888 $\times 10^{-22}$
gene1929	pta	MULTISPECIES: phosphate acetyltransferase	2.398631569	4.19040815291 $\times 10^{-22}$
gene0447	rpmJ	MULTISPECIES: 50S ribosomal protein L36	3.739631167	5.14192095846 $\times 10^{-22}$
gene5496	atpF	F0F1 ATP synthase subunit B	2.410464611	6.63432826519 $\times 10^{-22}$
gene1836	insB	insertion element IS1 protein insB	11.90886704	2.77662444484 $\times 10^{-21}$
gene4561	dnaE	DNA polymerase III subunit alpha	2.459924456	3.6441129141 $\times 10^{-21}$
gene0434	rpmC	MULTISPECIES: 50S ribosomal protein L29	4.119737978	1.46871803736 $\times 10^{-20}$
gene5090	phnJ	MULTISPECIES: alpha-D-ribose 1-methylphosphonate 5-phosphate C-P-lyase PhnJ	2.194323167	3.58026095948 $\times 10^{-19}$
gene5038	groEL	chaperonin Cpn60	3.37745451	9.68671395338 $\times 10^{-19}$
gene4235	ribH	MULTISPECIES: 6,7-dimethyl-8-ribityllumazine synthase	2.375219866	1.15312187355 $\times 10^{-18}$
gene1942	nuoE	MULTISPECIES: NADH-quinone oxidoreductase subunit NuoE	2.682849593	2.14343777835 $\times 10^{-16}$
gene4970	rpsR	MULTISPECIES: 30S ribosomal protein S18	2.438245493	2.8469474983 $\times 10^{-16}$
gene4726	lspA	Lipoprotein signal peptidase	2.303079148	1.19623566498 $\times 10^{-15}$
gene0444	rpmD	MULTISPECIES: 50S ribosomal protein L30	3.495310872	1.44067481979 $\times 10^{-15}$
gene0586	infB	translation initiation factor IF-2	2.037760507	3.13013293144 $\times 10^{-15}$

gene0589	rpsO	MULTISPECIES: 30S ribosomal protein S15	2.009046886	$4.46420181742 \times 10^{-15}$
gene0427	rplD	MULTISPECIES: 50S ribosomal protein L4	2.712545465	$6.24555206926 \times 10^{-15}$
gene0075	rpmB	MULTISPECIES: 50S ribosomal protein L28	2.214368589	$7.33331063716 \times 10^{-15}$
gene4634	aceE	pyruvate dehydrogenase (acetyl-transferring), homodimeric type	3.609179128	$7.55508464114 \times 10^{-15}$
gene3907	sdhB	MULTISPECIES: succinate dehydrogenase iron-sulfur subunit SdhB	3.596279784	$2.35108588685 \times 10^{-14}$
gene4566	fabZ	(3R)-hydroxymyristoyl-ACP dehydratase	2.186430344	$8.16821736247 \times 10^{-14}$
gene1021	zapA	MULTISPECIES: cell division protein ZapA	3.823708044	$8.1954390454 \times 10^{-14}$
gene4808	hpaE	5-carboxymethyl-2-hydroxymuconate semialdehyde dehydrogenase	3.945763791	$8.19935440857 \times 10^{-14}$
gene3036	zntB	MULTISPECIES: zinc transporter ZntB	3.043378595	$1.99371195492 \times 10^{-13}$
gene0636	glxK	MULTISPECIES: glycerate 2-kinase	4.608021625	$1.47623115119 \times 10^{-12}$
gene0076	rpmG	MULTISPECIES: 50S ribosomal protein L33	2.320911154	$3.34947012122 \times 10^{-11}$
gene4632	pdhD	dihydrolipoamide dehydrogenase	2.096329671	$4.37303748389 \times 10^{-11}$
gene3905	sucB	MULTISPECIES: 2-oxoglutarate dehydrogenase complex dihydrolipoyllysine-residue succinyltransferase	2.07501262	$1.00966030031 \times 10^{-10}$
gene4969	rplI	MULTISPECIES: 50S ribosomal protein L9	2.081661702	$1.61599390173 \times 10^{-10}$
gene2217	insB	IS1 family transposase	8.734479877	$3.40734657453 \times 10^{-10}$
gene0426	rplC	MULTISPECIES: 50S ribosomal protein L3	2.295533808	$8.82169531192 \times 10^{-10}$
gene4720	carA	MULTISPECIES: glutamine-hydrolyzing carbamoyl-phosphate synthase small subunit	2.287354502	$1.16287285112 \times 10^{-8}$
gene5161	malF	maltose transporter membrane protein	3.030143237	$1.74198012525 \times 10^{-8}$
gene1672	eutM	ethanolamine utilization microcompartment protein EutM	3.95876457	0.00032464760967
gene0290	livH	MULTISPECIES: high-affinity branched-chain amino acid ABC transporter permease LivH	3.322149495	0.000803529511878
gene1680	eutB	ethanolamine ammonia-lyase, large subunit	2.544458009	0.00319243627041
gene2148	gatY-kbaY	MULTISPECIES: tagatose-bisphosphate aldolase subunit GatY	2.847817862	0.0358727795107

Note: Only genes meeting the threshold of $|\log_2 FC| > 1$ and adjusted $p < 0.05$ are included. The full dataset is available upon request.

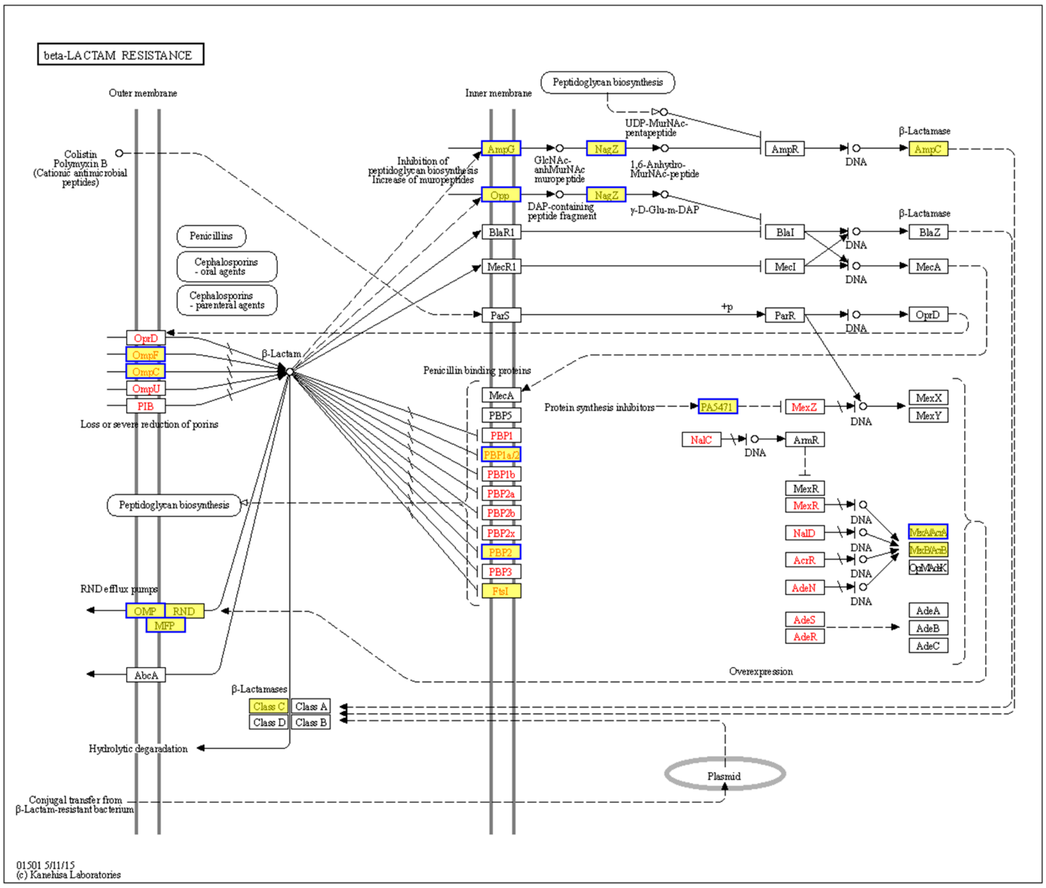

beta-LACTAM RESISTANCE
Outer membrane
Inner membrane
Peptidoglycan biosynthesis
Colistin
Polymyxin B
(Cationic antimicrobial peptides)
Penicillins
Cephalosporins - oral agents
Cephalosporins - parenteral agents
AmpG
NagZ
Opp
BlaR1
MecR1
ParS
AmpR
AmpC
BlaI
BlaZ
MecI
MecA
ParR
OprD
β-Lactamase
DNA
UDP-MurNAc-pentapeptide
GlcNAc-anhMurNAc muropeptide
1,6-Anhydro-MurNAc-peptide
DAP-containing peptide fragment
γ-D-Glu-m-DAP
Inhibition of peptidoglycan biosynthesis
Increase of muropeptides
+p
OmpF
OmpC
OmpU
PIB
Loss or severe reduction of porins
β-Lactam
Penicillin binding proteins
MecA
PBP5
PBP1
PBP1a/2
PBP1b
PBP2a
PBP2b
PBP2x
PBP2
PBP3
FtsI
Protein synthesis inhibitors
PA5471
MexZ
MexX
MexY
NalC
ArmR
MexR
NalD
AcrR
AdeN
MexA/AcrA
MexB/AcrB
OprM/TolC
AdeS
AdeR
AdeA
AdeB
AdeC
RND efflux pumps
OMP
RND
MFP
AbcA
Overexpression
β-Lactamases
Class C
Class A
Class D
Class B
Hydrolytic degaradation
Plasmid
Conjugal transfer from β-Lactam-resistant bacterium
01501 5/11/15
(c) Kanehisa Laboratories

(**A**)

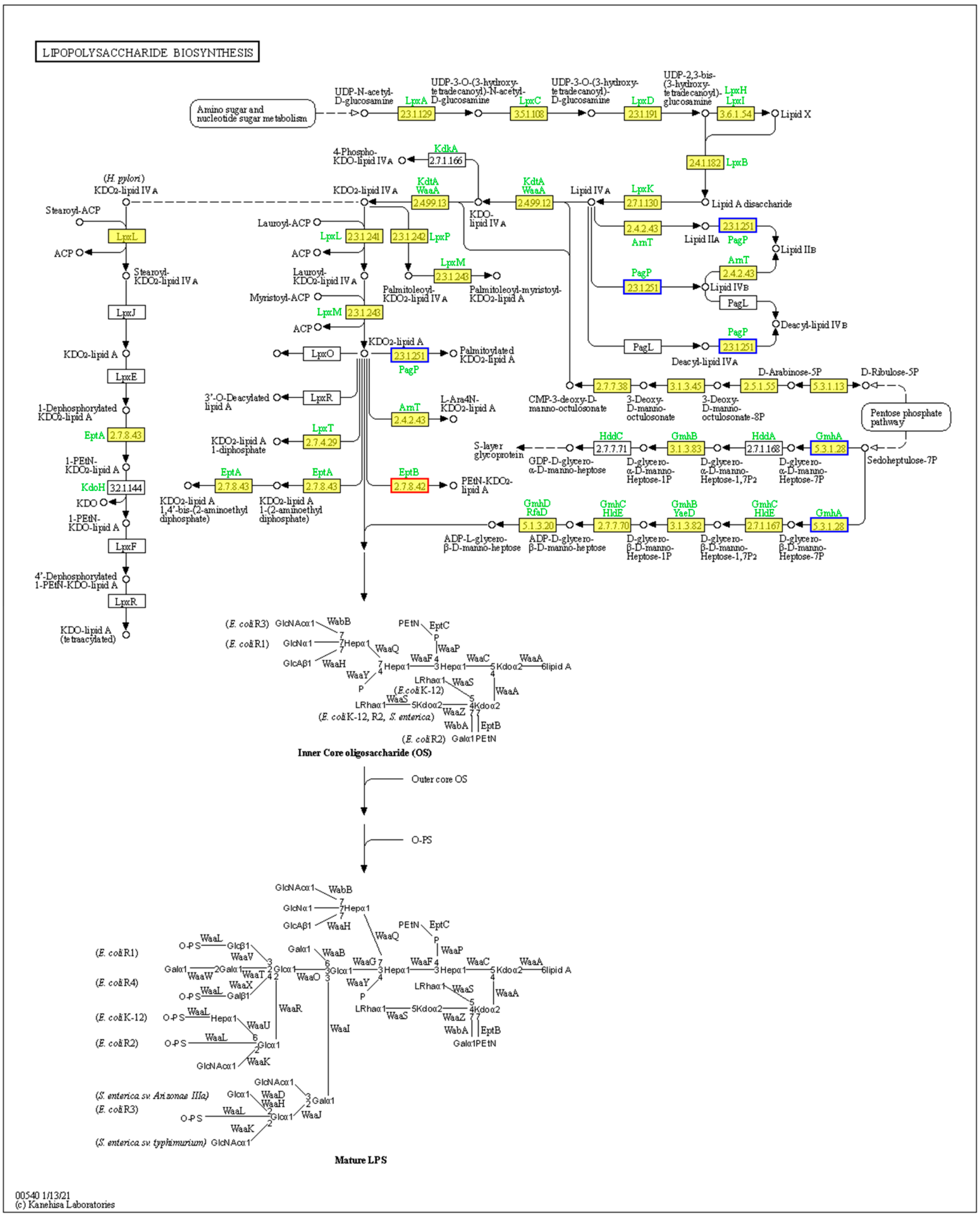
LIPOPOLYSACCHARIDE BIOSYNTHESIS
Inner Core oligosaccharide (OS)
Mature LPS
00540 1/13/21
(c) Kanehisa Laboratories

(**B**)

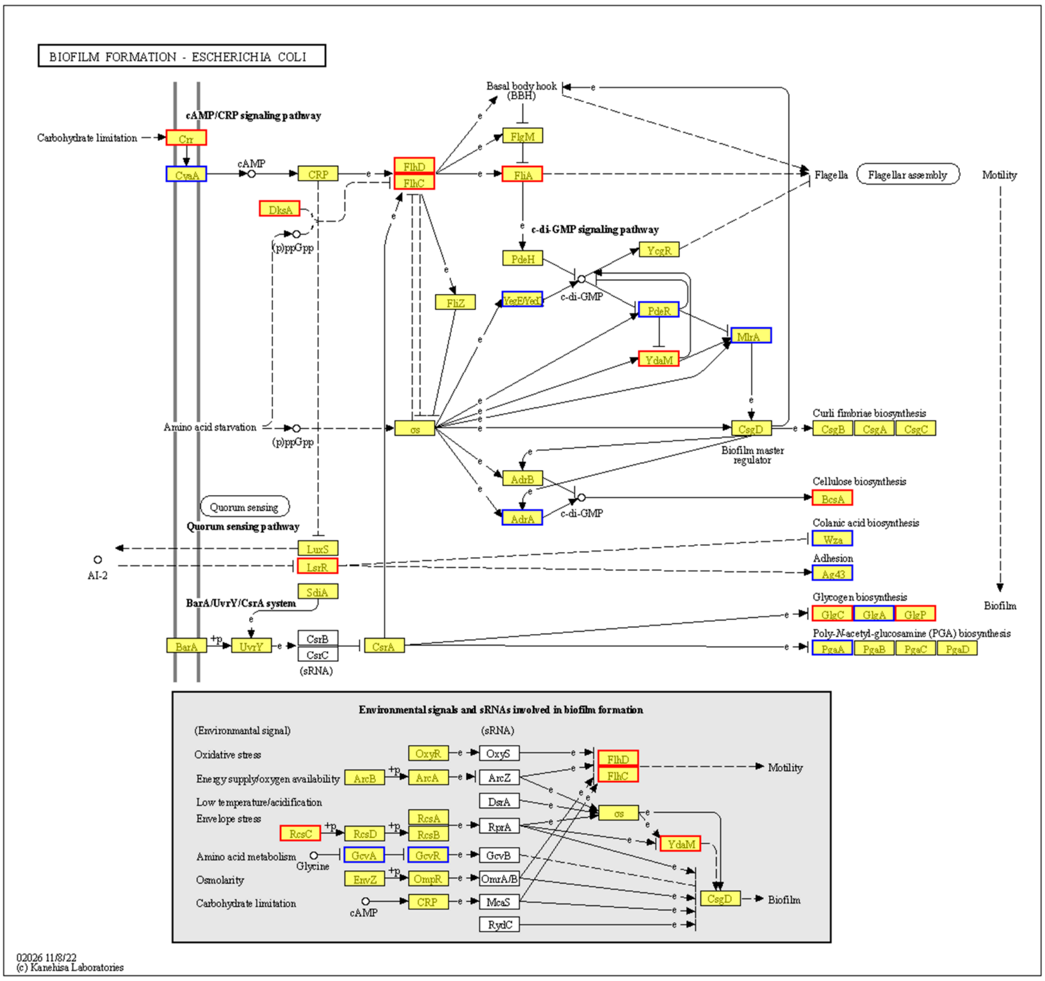
BIOFILM FORMATION - ESCHERICHIA COLI
cAMP/CRP signaling pathway
Carbohydrate limitation
Crr
CyaA
cAMP
CRP
FlhD
FlhC
DksA
(p)ppGpp
Basal body hook (BBH)
FlgM
FliA
Flagella
Flagellar assembly
Motility
c-di-GMP signaling pathway
PdeH
YcgR
FliZ
YegE/YedQ
c-di-GMP
PdeR
MlrA
YdaM
Amino acid starvation
(p)ppGpp
σS
CsgD
Curli fimbriae biosynthesis
CsgB
CsgA
CsgC
Biofilm master regulator
AdrB
AdrA
c-di-GMP
Cellulose biosynthesis
BcsA
Quorum sensing
Quorum sensing pathway
LuxS
LsrR
AI-2
Colanic acid biosynthesis
Wza
Adhesion
Ag43
SdiA
BarA/UvrY/CsrA system
Glycogen biosynthesis
GlgC
GlgA
GlgP
Biofilm
BarA
+p
UvrY
CsrB
CsrC
(sRNA)
CsrA
Poly-N-acetyl-glucosamine (PGA) biosynthesis
PgaA
PgaB
PgaC
PgaD
Environmental signals and sRNAs involved in biofilm formation
(Environmental signal)
(sRNA)
Oxidative stress
OxyR
OxyS
Energy supply/oxygen availability
ArcB
ArcA
ArcZ
FlhD
FlhC
Motility
Low temperature/acidification
DsrA
Envelope stress
RcsC
RcsD
RcsA
RcsB
RprA
σS
YdaM
Amino acid metabolism
Glycine
GcvA
GcvR
GcvB
Osmolarity
EnvZ
OmpR
OmrA/B
Carbohydrate limitation
cAMP
CRP
McaS
RydC
CsgD
Biofilm
02026 11/8/22
(c) Kanehisa Laboratories

(C)

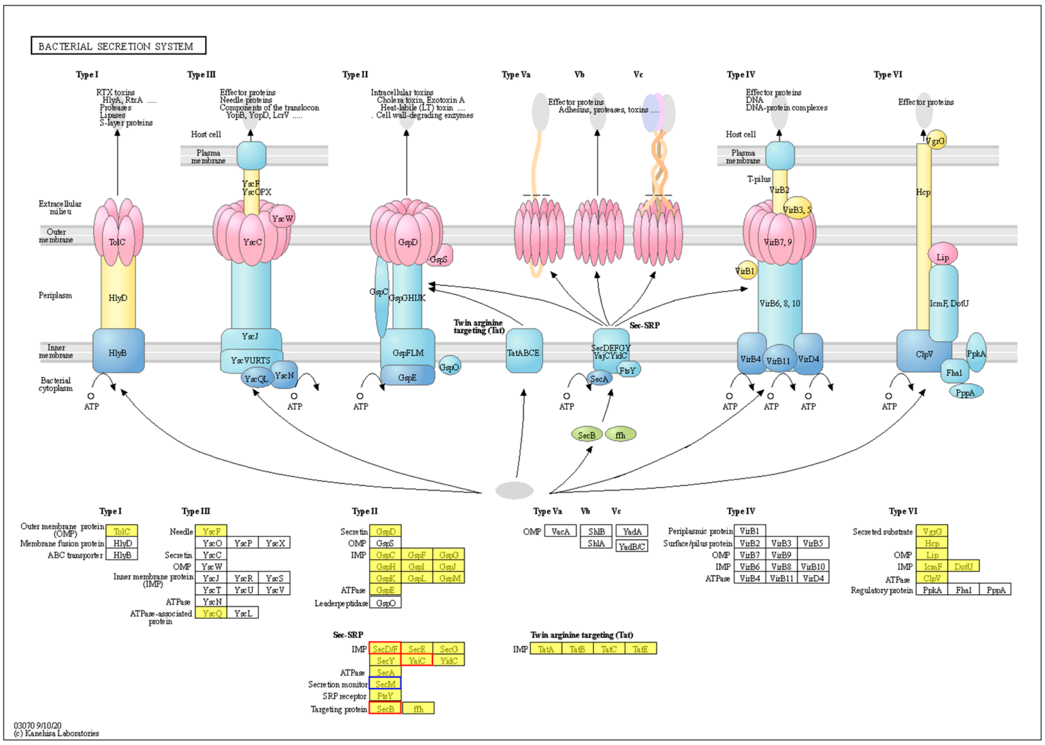

(**D**)

Figure S1. KEGG functional pathway diagram of significantly up-regulated and down-regulated differentially expressed genes. Notes: (**A**): β-lactam resistance pathway; (**B**): lipopolysaccharide biosynthesis pathway diagram; (**C**): biofilm formation pathway diagram; (**D**): bacterial secretion system pathway.
